# Introductory Lectures, No. 3

**Published:** 1848-05

**Authors:** 


ART. V.—*Introductory Lectures, No.* 3.—We mentioned at the close of the article on this subject in the last No., that we have been favored with copies a few introductories since the commencement of Art. No. 1. Not to be inattentive or invidious, with respect to these favors, we must again resume the subject.

The first which we will now notice is a "*lecture introductory to a course on obstetrics and the diseases of women and children.* By GUNNING S. BEDFORD, M. D., Prof. etc. in the University of New York." This is the only introductory from the city of New York which has reached us. The lecturer presents some of the considerations which render his province of instruction one of peculiar interest and importance to the student, and introduces several cases in illustration of his remarks. In the following extract, reference is made to errors in diagnosis, which are perhaps as frequent, as they are unfortunate. To distinguish between neuralgic affections of internal organs and the phlegmasiæ, is very necessary, not only for the success of medical treatment, but in order that injury may not be inflicted by inappropriate remedies :—

"The peculiar nervous susceptibility of the female not only occasions her great suffering, but frequently leads the practitioner into serious, if not fatal error. A lady, for example, has suffered for months from agonizing pain ; she consults her physician—she receives no benefit ; her medical attendant has committed the blunder of supposing the pain to originate in inflammation of some vital structure ; if the pain be in the chest, the *pleura* he imagines to be the seat of disease ; if in the abdomen, the peritoneum, liver, &c. Such cases not only bring discredit upon the profession, and expose it to the severe criticisms of the skeptical in medicine, but they oftentimes entail on the suffering patient years of unnecessary anguish. I cannot do better than quote on this subject the following remarks of a modern writer, who is not only a man of profound erudition, but one who is bold enough to think for himself. "Next to the suspicion of peritonitis," he observes "the belief that some individual organ in the abdomen is inflamed, has been among the most frequent errors in cases of hysteric pain ; again and again have I seen complaints of this kind treated as chronic hepatitis ; and the disease confirmed, and the constitution undermined by the use of mercury. On other occasions, when pain on the left side had been accompanied by irregular hurried action of the heart, which is very frequent, the pericardium has been supposed to be inflamed, or organic disease of the heart suspected." All practitioners of experience will confirm the truth of these remarks, and bear testimony to the fatal errors constantly occurring in practice from culpable neglect of what really constitutes the very foundation of all successful treatment, accuracy in *diagnosis*."

The following case, cited by the lecturer in illustration of the sympathetic morbid influences emanating from the uterus, has given rise to some severity of criticism. The introduction of the names of medical gentlemen who had previously been consulted in the case, and the implication of their judgment, in connection with the implied superiority of tact and skill in the reporter, has been commented on in some of our contemporaries as improper and unprofessional. Another feature of this portion of the discourse has, also, been censured, viz: the exposition of the method of procuring abortion by mechanical means. The latter objection involves the fact that the discourse was published in a popular newspaper in the city of New-York, as has been intimated, with the author's consent. To the latter insinuation Prof. B. has entered a positive disclaimer, and, this being admitted, we are at a loss to see any impropriety in the passage objected to. The discourse was addressed to medical students, and was published, as is to be presumed, for the benefit of medical students and practitioners. If such things are objectionable in a publication of this character, they are equally so in medical or surgical treatises. Besides, it is in vain to expect to keep mankind in ignorance of the means of doing evil; the prevention of evil deeds is not thus to be attained. With reference to the criticism first mentioned, it seems to us to be merited, but we are bound in charity to believe that the author was betrayed into the offence by inadvertency. The case detailed in the extract referred to is interesting, and illustrates in a striking manner the point, for the exemplification of which it is submitted by the lecturer. It is as follows:—

"The practitioner who desires to treat successfully the maladies incident to the female, must constantly bear in mind the numerous sympathies, by which the womb is allied to almost every portion of the system. The life of the patient, indeed, often depend on this knowledge. How often, for example, does it occur that the first indication of disease in the uterus is exhibited, not by any local disturbance of that organ, but by the manifestation of some of the numerous sympathetic phenomena. I have been repeatedly consulted by ladies who have suffered severely for months from local pain in the stomach, kidneys, liver, or some other important organ. They have had various remedies applied, having been treated for disease of the liver, kidneys, &c., but without benefit. On a minute investigation of their case, I have found disease of the neck of the womb, sometimes slight, sometimes of an aggravated character, to be the sole cause of their suffering. In restoring the womb to a healthy condition their pains at once subsided.

"The following case will illustrate forcibly the absolute necessity of constantly bearing in mind the influence exerted by the womb in certain forms of disease: During the month of February last, I was requested to visit a lady in consultation with Dr. Whiting of this city. Several medical gentlemen, among whom was Dr. Willard Parker, had, previous to my visit, seen and prescribed for this patient. When I saw her in company with Dr. Whiting, she was apparently near dissolution. Her prostration was extreme; her countenance almost hippocratic; in a word, her friends had abandoned all hope of recovery. The particulars of the case were these: she was the mother of one child, seventeen months old. About a month previous to my seeing her, she had occasionally been troubled with nausea and vomiting, and for the week previous to my visit she had vomited incessantly. She could retain nothing on her stomach; the vomiting resisted every remedy that had been administered. It was under these circumstances that I was called to her. The medical gentlemen who had preceded me in attendance, ordered cups, leeches, and blisters over the region of the stomach, with various internal remedies, but all without the slightest appreciable effect. The vomiting was still unchecked, and the death hourly expected. On examining critically her case, I arrived at the conclusion that the vomiting was merely a symptom of trouble elsewhere, and that no remedy which could be addressed to the stomach would be of the least avail in rescuing her from the imminent peril in which she was placed. In putting my hand on the abdomen, I found the uterus was enlarged, and occupying the hypogastric region. The alarming situation of the patient would not justify delay; if her life was to be saved, every thing admonished us that it was to be done only by instantaneous measures. My opinion of the case was, that the vomiting was sympathetic, produced by irritation of the womb. I therefore suggested the propriety of endeavoring to induce contraction of this organ, in order that its contents might be expelled. This view was concurred in by Dr. Whiting. Accordingly, without a moment's delay, desperate and almost hopeless as the case was, I introduced a female catheter, into the womb, and ruptured the membranes; in a short time the uterus contracted, and a mass of hydatids was thrown off. Immediately, as if by enchantment, the vomiting ceased. The patient, after a tedious convalescence from her extreme debility, recovered, and is now in the enjoyment of robust health.

"Let this case, gentlemen, impress on your minds the importance of tracing effects to causes; and remember this cardinal truth—that the practitioner who prescribes for mere symptoms can never hope successfully to treat disease."

The lecture, as a whole, is calculated to impress the mind of the reader with a favorable opinion of the abilities of the author as a writer and teacher. It closes with a feeling tribute to the memory of the late Prof. Revere.

*Address at the opening of the session of* 1847–8, *of the Indiana Medical College.* By E. Denning, M. D., Professor of Materia Medica. Published by the class.

The subject of this discourse is medical education. The author insists on "the necessity of creating and sustaining a higher standard of sound medical education." He advocates the importance of educational institutions established in different sections of the country. It is needless for us to say, that, in our humble opinion, this is sound doctrine. Different locations for medical schools, doubtless, differ greatly as respects local advantages, nor do we deny that the number of schools may be unduly multiplied. But that the medical sciences are to be taught, forsooth, in two or three places only, seems to us a preposterous absurdity. To exclude emuation among schools would be to cause medical education to recede, instead of advancing. In proportion, too, as medical instruction is concentrated, would the influence of individual authority be likely to affect injuriously medical opinions. We quote as follows :—

"In the attainment of a high standard of medical education, and sound medical literature, above all others, important responsibilities rest on the American students of medicine.

The circumstances of our country, and the genius of our institutions, give to the American mind extraordinary facilities for improvement. Our system of common schools, and other means of intellectual and moral elevation, afford ample means for laying early and solid foundations for the acquisition of elementary knowledge, preparatory to professional study. To the medical student, the extent and variety of climate and soil, temperature and botanical productions, together with diseases of the different portions of the country, an almost boundless field is opened, and if he enters it with proper training, with the determination to make all these means subservient to the attainment of truth, and facts relative to his profession—if he but make his mind the great store-house, well filled and arranged, and classified in order, he will be enabled to go forth in the consciousness of possessing such qualifications as will enable him to administer relief to human suffering,

To perfect these means, and render them available as remedial agents, well regulated medical colleges are indispensable, and without consuming time in the argument, I assume it as an important fact, that their location should have some relation to the peculiar diseases of the district surrounding them. With this view, it seems to me important that the north west should sustain its institutions. What great good or superior advantage does a student gain by attending medical courses east? Let us for a moment examine. Is man differently constituted there? No, say you, he is every where the same. Well, then, the knife can here reveal the same brain, heart, lungs, stomach, liver, viscera, nerves, bloodvessels and the like. If the human structure is the same here, then here is the place to study it. Are not the laws of chemistry the same here as there? While natural "philosophy teaches the action of bodies one upon another as wholes, chemistry goes into particulars," and shows how these bodies act upon one another by particles, and in that manner covers the whole ground. Is the book of nature any larger there than here? Is the action of therapeutical agents in the pathological condition different there from this region? Do they know any more of the action of antimony, mercury, iodine, and quinia, than we do? If not, then sustain our own institutions. Cannot the great operations of surgery be as well performed and demonstrated here as there? Are not the operations of amputation, lithotomy, tying an artery, the same here as at the east? Is not every thing relating to obstetric medicine as well understood here as there? Are not the means of instruction as ample?

In the history and treatment of disease, there is no essential deficiency. The great lights of the past shine as brilliantly here as in trans montane regions. The general causes of the alteration of structure and derangement of function are as clearly understood. In addition to all this, the advantage of attending to the specific history and treatment of western disease is of great importance. These are clearly defined—their pathological condition is better understood—the remedial agents with their qualities and doses are adapted to the abnormal state—their special adaptation to the diseased functions has been more clearly delineated. All this has been obtained by observation and experience."

We have still another introductory from the Indiana Medical College. It is thus entitled:—"*A Lecture on Atresia Vaginæ.* By N. Hard, M. D., Professor of Obstetrics, &c. Published by the Class." The larger portion of this discourse is taken up with the report of an interesting case of occlusion of the vagina and os uteri, which came under the author's observation. After retention and accumulation of the menses for five months within the cavity of the uterus, an artificial conduit was made by a surgical operation, in which it became necessary to penetrate, with the bistoury and trochar, the uterus, as well as the obliterated vagina. The occlusion was produced by sloughing of the parts after a tedious, instrumental labor. The case is valuable, and, but for its length, we should transfer it to our columns. It should be added that the operation was successful, the patient having since menstruated through the artificial orifice into the uterus.

"*An Introductory Address on the life and character of the late Prof. Noah Worcester, M. D.* By Jacob C. Delamater, M. D., Prof. of Anat. and Phys. Published by the Medical Class."

This is the title of an introductory delivered by Prof. Delamater, jr., before the medical class at Cleveland, Ohio. In the decease of the late Prof. Worcester the profession of the country sustained the loss of one of its brightest ornaments. He was stricken down in the midst of a career of honor and usefulness. Possessing rare scientific attainments, intellectual abilities of the first order, and those moral qualities which are so important elements in the character of the truly excellent practitioner and teacher, he was destined, had Providence spared his life, to have attained a position among the highest in the ranks of our profession. But, what is of far greater importance than professional eminence, in his life were exemplified those virtues and graces which characterize the good citizen, the gentleman, the true friend, and the christian. In the varied relations of life his was an example worthy of imitation. The biographies of such men are not only replete with interest, but exert upon society a healthful, invigorating moral influence. The history of their lives encourages and instructs after their bodies have ceased to live. To honor their names is not only a just tribute to their memories, but a duty to ourselves and our race, inasmuch as we, and others, may be thereby incited to follow in their footsteps.

We commend the lecture of Dr. Delamater most cordially to our readers. The author has been at pains to collect data for a highly satisfactory biography of his late colleague.

We select for quotation the portion which gives an account of his last moments : —

"He died young—before the sands of his 36th year had all run out. He died a martyr to the severe toils of professional life, and the wearing influence of deep affection daily sundered by death. His wife, his mother, two well-beloved sisters, and his respected father went to rest before him.

When the report was circulated that the life of Prof. Worcester was at hazard, deep anxiety and gloom sat upon many countenances, and from that time onward, the messages of enquiry and personal offers of kindness in any form in which it could be available, were numerous and constant.

But while others were manifesting alarm and sorrow, he was calm and resigned to the will of Providence.

He conversed freely with his friends on the subject of Death, expressed his deep regret that he had not made a public profession of religion, remarking that it was a great source of sorrow that he had permitted a few unimportant matters to prevent him from uniting with the church. He exclaimed, "Oh, how trifling they appear to me now, upon a bed of death, and I pray God to forgive me this great sin."

He expressed a full belief in the doctrines of the Bible, hoping only for salvation through Christ.

During his closing hours, he remarked to Professor Mussey, who was much with him, that he had three friends in the Medical Profession, whom he had loved and valued above all others; and added, "it will not be long before I may hope to meet them in Heaven." These had all, at some period of his life, been his instructors.

As he drew near to death, his sense of gratitude to God, was strong, and his faith triumphant. "How good" said he, "has God been to our family, and especially to myself. I have been happy nearly all my life, and yet *the happiest hour is the hour of my death.* I feel no pain, no care, nothing but the love and presence of God," and he died with the words on his lips, "Lord Jesus, receive my spirit." His short and brilliant career was ended."

*An Introductory Address, delivered at the opening of the session of* 1847–8, *to the Students of the Memphis Medical College, Nov.* 1, 1847. By George R. Grant, M. D., Prof. of the theory and practice of Medicine, in the Memphis Medical College.

This introductory comes to us in the *South-Western Medical Advocate,* a medical journal recently established at Memphis, edited by James Conquest Cross, M. D., one of the Faculty of the Memphis School.

The Memphis School is of recent date. Prof. Grant's discourse was an introductory at the opening of its second session, and the charter of this institution is the first ever granted by the State of Tennesee for medical instruction. We learn, also, from the address that in Tennesee, as has been generally the case in other States, the only aid granted by the State to the Institution, consisted in the act of incorporation. The State of New-York has, we suspect, been more liberal in pecuniary appropriations for the benefit of medical Colleges than most of her sister States, notwithstanding her erratic course of legislation with regard to the medical profession in other respects.

Dr. Grant devotes considerable space to the discussion of the proposition, "*that the profession ought to be studied near by the region where it is designed to be practiced.*" This proposition has been for some years a bone of contention between some of our Southern and Northern brethren. Its *practical* bearings are sufficiently obvious. For ourselves we must incur the risk of being included among those whom the writer styles "pseudo-critics,"

and charges with "arrogance and presumption," inasmuch as the proposition appears to us, to say the least, to merit far less importance than is attached to it by some of our Southern and Western friends. So far from its being essential to prosecute studies at, or near the localities where the student expects to perform the practical duties of the profession, in our opinion the practitioner who emigrates to a new section of country, has, in many instances, some positive advantages over his indigenous brethren. If he be well educated, he is certainly not less qualified to observe and study endemic diseases, and endemic traits of disease, than the student before graduation; and if he have a practical acquaintance with the peculiarities of disease in other localities, he is able to avail himself of the comparison between the phenomena which he has seen, and those now before him. It is obvious his progress must be more rapid, conceding that the diseases of different localities require separate study—which, to a certain extent, we are free to admit. But in another point of view he has an advantage—he is less likely to imbibe local prejudices, and those errors of experience which are based upon *false facts*, and perpetuated by individual authority. He is, in short, a more impartial, and, consequently, a more accurate observer, admitting him to possess talents and acquirements no more than equal with those among whom he has come to reside. We are sorry to see this taken as the ground upon which a school advocates it claims to support. It is partial, untenable, and needless. Let those engaged in building up the Memphis College make a good school, and rest its claims for success wholly upon its merits. We believe that Prof. G. is right in assuming that there is no impracticability in undertaking that the various departments of medical science shall be as well taught at Memphis as elsewhere. Let this be done, and let the profession see that it is done, and we know not why any meretricious claims for sectional support should be found requisite. We are gratified to learn that the Institution has commenced its existence under very encouraging auspices, and promises to realize the wishes of its founders. We quote the peroration of the discourse, in which the lecturer appeals to the *southern patriotism* (if the solecism is admissible) of his auditory:—

"Remember that you are *Southerners*. Nature has ever been lavish of her gifts in such localities. Here she is seen in the fullness of her majesty, as displayed in the physical world around us:—In the magnificence of our forests; in the productiveness our soil; and in the length and breadth of our "inland seas."

Nor is she less prolific in the development of MIND. Our our statesmen, our soldiers, our scholars, have ever been, when occasion required, or duty called, among the first in the forum—with the bravest on the battle-field—and second to none where science, or literature was the object of pursuit.

From you, then, whose physical conformation has been matured beneath a Southern sun; and whose mental growth has been strengthened by the education furnished at Southern Institutions of learning, science expects *much*; and with those whom I now have the pleasure of addressing, I feel every assurance that her requirements will prove an easy, and a pleasing task.

The new era which is dawning upon Medicine, will be succeeded by a meridian splendor of scientific achievements, a large supply of which, from the very nature of things, should be derived from the contributions furnished by *Southern intellect.*"

The last of our present stock of introductory addresses is contained in the *New Orleans Medical and Surgical Journal*, and was delivered by John Harrison, M. D., Prof. of Physiology and Pathology in the University of Louisiaana. It was published in the New Orleans Journal by request of the class. This is decidedly one of the best introductories of the season. It is strictly speaking, introductory to the subjects which the author's course of lectures embraces. It is to the point; "sensible," without "dullness," and without "glittering wordiness," albeit it was delivered on the "banks of theMississippi." In reading the address, in order to select a portion for quotation, we find its unity of character to be such, that from a detached fragment the reader would form an inadequate opinion of the entire production. We prefer, therefore, to leave its symmetry undisturbed.

